# The role of erlotinib and the Optune device in a patient with an epidermal growth factor receptor viii amplified glioblastoma

**DOI:** 10.1093/omcr/omy095

**Published:** 2018-11-05

**Authors:** Sean P Doyle, Saumya S Gurbani, Alexandra S Ross, Havi Rosen, Charlice Dunn Barrett, Jeffrey J Olson, Hyunsuk Shim, Hui-Kuo Shu, Soma Sengupta

**Affiliations:** 1 Emory University School of Medicine, Atlanta, GA, USA; 2 Departments of Neurology and Medical Oncology, Emory University, Atlanta, GA, USA; 3 Department of Medical Oncology, Emory University, Atlanta, GA, USA; 4 Department of Neurosurgery, Emory University, Atlanta, GA, USA; 5 Department of Radiation Oncology, Emory University, Atlanta, GA, USA

## Abstract

The standard treatment for patients diagnosed with glioblastoma is surgical resection of tumor followed by high dose radiation and chemotherapy with temozolomide. For patients who experience allergic reactions to temozolomide despite desensitization protocols, alternative therapies must be considered. In this report, we present such a patient who then received treatment with an epidermal growth factor receptor inhibitor, erlotinib, concurrent with a tumor-treating field device, Optune. Through this combination of a targeted molecular therapy and the Optune device, the patient has been able to achieve stable disease 9 months after completing radiation.

## INTRODUCTION

Temozolomide (TMZ) is part of the standard chemotherapy regimen for patients with newly diagnosed glioblastomas (GBMs) [1]. If a patient experiences an allergic reaction to TMZ, a desensitization protocol is implemented [2]. If this fails, other chemotherapeutic agents must be considered. In this case report, a patient who was unable to sustain multiple cycles of TMZ is treated with an epidermal growth factor receptor (EGFR) inhibitor in combination with tumor-treating electric fields (TTFs) and has been able to achieve a stable disease course.

## CASE REPORT

**Figure 1:**
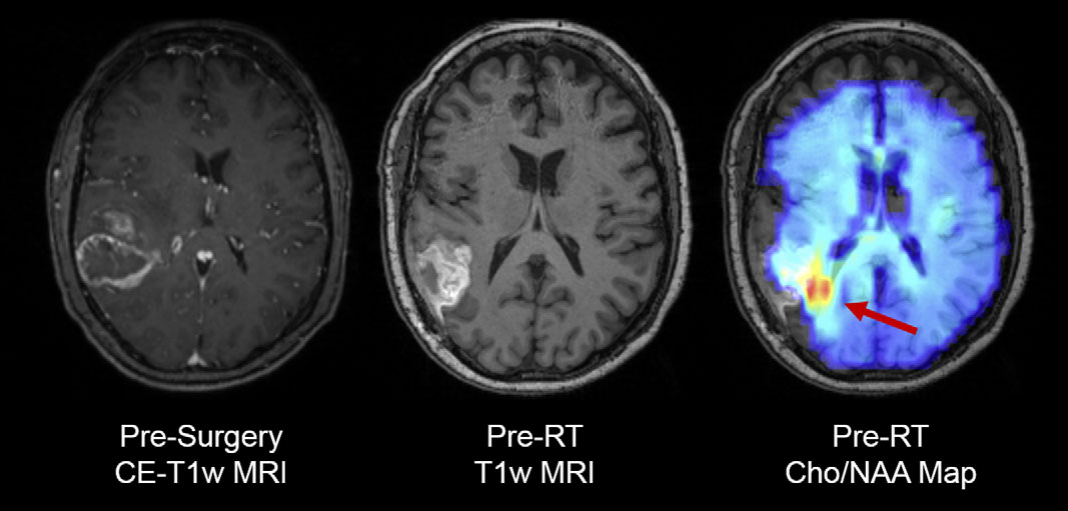
Contrast-enhanced T1-weighted MRI (CE-T1w MRI) indicated a high-grade brain tumor at the time of diagnosis, which was surgically resected and confirmed to be glioblastoma. Prior to starting chemoradiation, spectroscopic MRI showed an elevated choline to *N*-acetylaspartate (Cho/NAA) lesion medial to the resection cavity, indicating the presence of residual active tumor (red arrow)

A 66-year-old woman presented with polyuria and polydipsia for 3 weeks prior to evaluation by her primary care physician. Because of the concern for diabetes insipidus, the patient underwent MRI of the brain with and without contrast. Scans showed a right temporoparietal brain lesion ~4.5 cm × 4 cm in size (Fig. 1). The patient underwent surgical resection, and the neurosurgeons achieved gross total resection with an absence of visible disease on contrast-enhanced MRI. A diagnosis of GBM was made and testing determined the tumor to be methyl guanine methyl transferase (MGMT) hypermethylated, *EGFR* amplified and EGFRviii positive.

**Figure 2:**
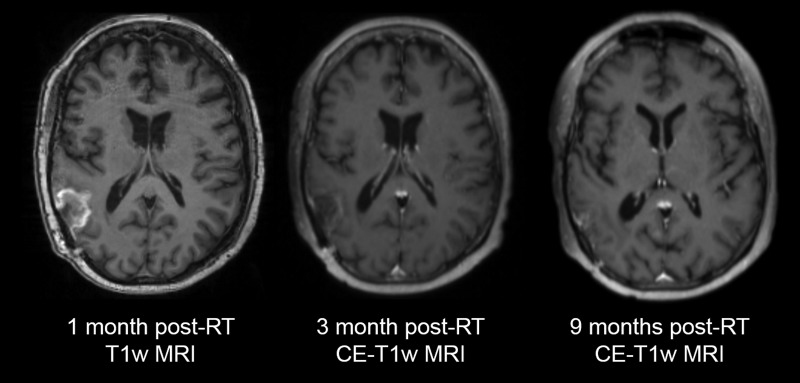
The patient’s disease has been stable for a period of 9 months post-radiation therapy (RT) via maintenance therapy consisting of erlotinib + Optune after the patient was removed from the standard TMZ regimen due to a hypersensitivity reaction

The patient opted to enroll in a clinical trial that uses 3D spectroscopic MRI [3] to monitor the metabolic response of patients to an experimental histone deacetylase inhibitor (HDACi), belinostat, concurrent with TMZ and radiation therapy. Pan-isoform HDACi’s like belinostat are hypothesized to have a synergistic effect with TMZ for radiosensitization of tumor cells; belinostat has more blood–brain barrier penetration than other HDACi’s [4]. A post-resection sMRI scan suggested the presence of residual non-enhancing disease. She received 60 Gy radiation in 30 fractions over 6 weeks in conjunction with belinostat and TMZ, and appeared to have stable disease per MRI 1-month post-radiation (Fig. 2).

One month later, the patient experienced linguofacial swelling and hives after her first cycle of adjuvant TMZ. Recognizing this to be an allergic reaction, an extensive desensitization regimen was performed to no avail. The patient was taken off the clinical study and alternative chemotherapeutic agents were considered. Since the patient could not tolerate TMZ and refused to try other alkylating agents, and noting that her tumor exhibited mutated *EGFR*, she was started on erlotinib, an EGFR inhibitor used primarily in treatment of non-small cell lung cancer [5]. Concurrently, she began use of Novocure’s Optune, a device which generates low intensity TTFs via a scalp-mounted transducer array [6]. The patient has tolerated the treatment well and her complaints thus far have been left arm paresthesia while playing stringed instruments, as well as scalp, face and arm irritation from the Optune device. She is on a regimen of 7× 150 mg tablets of oral erlotinib weekly. Per imaging and clinical course, she appears to have stable disease 9 months post-radiation (Fig. 2).

## DISCUSSION

Standard of care for patients with GBM consists of maximal safe tumor resection followed by radiotherapy with concurrent and then adjuvant TMZ. Virtually all patients that receive this multifaceted treatment regimen eventually experience disease progression, resulting in a median overall survival of only 16–19 months [1]. Recognizing the need for improved treatments for GBM, recent clinical trials have evaluated the efficacy of intermediate-frequency TTFs as an additional maintenance therapy [7]. In these clinical trials, overall survival was observed to improve by a median of 5 months when TTFs were included in combination with radiotherapy and TMZ. Although TTFs are now clinically employed as a component of GBM maintenance therapy along with TMZ, use of TTFs in combination with other molecularly targeted GBM therapies has yet to be evaluated. An assessment of such alternative therapeutic strategies is greatly needed for patients who are unable to tolerate the standard-of-care TMZ regimen, such as in the case of the patient presented in this report.

Recent efforts have been made to sequence the exomes of GBMs to identify prognostic markers and promising therapeutic targets. Two commonly recurring genetic lesions in GBM are amplification of *EGFR* and deletion of *EGFR* exons 2–7, which results in the generation of a constitutively active EGFRviii variant that drives tumor proliferation. Molecular profiling of this patient’s tumor revealed both *EGFR* amplification and deletion of exons 2–7, indicating that her tumor may be driven by overactivation of *EGFR*-related cell signaling pathways. While clinical trials evaluating the efficacy of EGFR tyrosine kinase inhibitors (TKIs, e.g. erlotinhib) in the treatment of EGFR-driven GBM have demonstrated no overall patient survival benefit, potentially because of the poor CNS penetration of TKIs [8], recent studies have shown efficacy of anti-EGFR antibodies conjugated to cytotoxic drugs as a vehicle for EGFR-directed therapies [9].

To date, few studies have examined the combined use of TTFs and targeted molecular therapies in the maintenance therapy stage of GBM treatment. Here, we report the use of a targeted EGFR inhibitor, erlotinib, in combination with TTFs in the maintenance therapy of a patient’s GBM tumor following maximal surgical resection. This therapeutic combination, initiated as an alternative therapy because of the patient’s hypersensitivity to TMZ, has resulted in stable tumor size and disease course for 9 months following completion of radiotherapy. While the exact role of erlotinib in this patient’s treatment outcome is unclear, prior surgical resection has resulted in significant blood–brain barrier disruption that allows for enhanced CNS uptake of therapeutic agents. It has also been hypothesized that TTFs may be able to further improve CNS penetrance of therapeutic agents [10], though this would need to be examined by future studies.

To our knowledge, this is the first report of combining erlotinib with the Optune device. We propose that further clinical trials that evaluate the use of targeted molecular therapies in combination with tumor-treating fields may be warranted, particularly for patients that are unable to tolerate standard TMZ chemotherapy.
